# Silica and asbestos exposure at work and the risk of bladder cancer in Canadian men: a population-based case-control study

**DOI:** 10.1186/s12885-020-6644-7

**Published:** 2020-03-03

**Authors:** Lidija Latifovic, Paul J. Villeneuve, Marie-Élise Parent, Linda Kachuri, The Canadian Cancer Registries Epidemiology Group, Farah McCrate, Ron Dewar, Nancy Kreiger, Donna Turner, Shelley A. Harris

**Affiliations:** 1 Occupational Cancer Research Centre, Cancer Care Ontario, Ontario Health, 525 University Ave, Toronto, ON Canada; 2 Dalla Lana School of Public Health, University of Toronto, 155 College St, 6th floor, Toronto, ON M5T 3M7 Canada; 3 School of Mathematics and Statistics, Carleton University, 1125 Colonel By Drive, Ottawa, ON Canada; 4 Centre Armand-Frappier Santé Biotechnologie, Institut national de la recherche scientifique, 531 boul des Prairies, Laval, QC Canada; 5 Department of Epidemiology & Biostatistics, University of California at San Francisco, San Francisco, CA USA

**Keywords:** Bladder cancer, Silica, Asbestos, Case-control study, Expert assessment, Occupational cancer risk factors

## Abstract

**Background:**

Silica and asbestos are recognized lung carcinogens. However, their role in carcinogenesis at other organs is less clear. Clearance of inhaled silica particles and asbestos fibers from the lungs may lead to translocation to sites such as the bladder where they may initiate carcinogenesis. We used data from a Canadian population-based case-control study to evaluate the associations between these workplace exposures and bladder cancer.

**Methods:**

Data from a population-based case-control study were used to characterize associations between workplace exposure to silica and asbestos and bladder cancer among men. Bladder cancer cases (*N* = 658) and age-frequency matched controls (*N* = 1360) were recruited within the National Enhanced Cancer Surveillance System from eight Canadian provinces (1994–97). Exposure concentration, frequency and reliability for silica and asbestos were assigned to each job, based on lifetime occupational histories, using a combination of job-exposure profiles and expert review. Exposure was modeled as ever/never, highest attained concentration, duration (years), highest attained frequency (% worktime) and cumulative exposure. Odds ratios (OR) and their 95% confidence intervals (CI) were estimated using adjusted logistic regression.

**Results:**

A modest (approximately 20%) increase in bladder cancer risk was found for ever having been exposed to silica, highest attained concentration and frequency of exposure but this increase was not statistically significant. Relative to unexposed, the odds of bladder cancer were 1.41 (95%CI: 1.01–1.98) times higher among men exposed to silica at work for ≥27 years. For asbestos, relative to unexposed, an increased risk of bladder cancer was observed for those first exposed ≥20 years ago (OR:2.04, 95%CI:1.25–3.34), those with a frequency of exposure of 5–30% of worktime (OR:1.45, 95%CI:1.06–1.98), and for those with < 10 years of exposure at low concentrations (OR:1.75, 95%CI:1.10–2.77) and the lower tertile of cumulative exposure (OR:1.69, 95%CI:1.07–2.65). However, no clear exposure-response relationships emerged.

**Conclusions:**

Our results indicate a slight increase in risk of bladder cancer with exposure to silica and asbestos, suggesting that the effects of these agents are broader than currently recognized. The findings from this study inform evidence-based action to enhance cancer prevention efforts, particularly for workers in industries with regular exposure**.**

## Background

Both silica and asbestos are widespread in the natural environment and present in low concentrations in ambient air. Silica is a metal oxide that exists in both crystalline and amorphous forms and is a major component of sand, rock, and mineral ores. It is one of the most prevalent occupational exposures worldwide with high proportions of exposed workers in occupations involving movement of the earth, such as mining, farming, quarrying, as well as construction, masonry, sandblasting, and production of glass, ceramics, and cement [1]. There are tens of millions of exposed workers worldwide [2]. An estimated 380,000 workers are exposed in Canada [3], 2.3 million in the U.S. [4], 3.2 million in Europe [5], more than 23 million in China [6] and over 10 million in India [7]. Asbestos is a fibrous silicate mineral found in metamorphic rock formations around the world. Historically, workers in mining, milling and those manufacturing asbestos products represented occupational populations with the highest levels of exposure; however, the relative contribution of these sources to asbestos exposure in the Canadian population is decreasing due to local mine closures and a 2018 federal ban on use. In recent years, over 60 countries have instituted national bans on the use of all types of asbestos; however, due to its historically widespread use in building construction, insulation, automotive parts, ship and boat building and textiles it is still a common occupational exposure today. Asbestos exposure occurs in the construction industry and related trades, from the repair, renovation, and demolition of older (pre-1980) buildings. Approximately 125 million people are exposed worldwide [8], with an estimated 152,000 Canadians exposed to asbestos at work [9]. Inhalation is the most common route of occupational exposure to both silica and asbestos [3, 9]. The latter are both recognized as human carcinogens. The International Agency for Research on Cancer (IARC) has classified inhaled crystalline silica as a human carcinogen based on a strong exposure-response relationship and an overall effect of silica on lung cancer [1]. Similarly, all forms of asbestos are recognized human carcinogens by IARC, the U.S. Environmental Protection Agency and the U.S. Department of Health and Human Services based on unequivocal epidemiologic evidence for lung cancer and mesothelioma [8, 9]. However, the impact of these exposures on the risk of cancer at other sites remains unclear.

While extra pulmonary translocation mechanisms of inhaled particles and fibers are not fully understood, the clearance of ultra-fine silica particles and small-diameter asbestos fibers from the lungs may lead to their dissemination and persistence at other organ sites [2, 10]. Particle size and physico-chemical properties determine particle clearance from the lungs. Smaller particles (< 2.5 μm) can penetrate more deeply and reach the alveoli and may be moved across the respiratory epithelium to alveolar-capillaries. This can lead to systemic dissemination to other organ sites [11] such as the bladder.

Bladder cancer is the ninth most common cancer worldwide and the sixth most common cancer among men worldwide with an estimated 430,000 new cases diagnosed in 2012 [12]. Urothelial carcinoma is the most common subtype of bladder cancer accounting for almost 90% of all bladder cancers [13]. Smoking is the most important risk factor for bladder cancer based on an attributable risk of 50% [14]. Other established risk factors include older age, male gender, exposure to arsenic in drinking water [15] and medical conditions such as chronic urinary retention and infection with schistosomiasis [14, 16]. Inherited genetic factors, such as slow acetylator N-acetyltransferase 2 variants, glutathione S-transferase mu 1-null genotypes and several other common sequence variants may increase susceptibility to carcinogens [17], mainly tobacco smoke [14]. Work-related exposures account for 1–8% of bladder cancer [18, 19]. This attributable risk is higher in occupations such as metal working, machining, transport equipment operators and miners [19]. Occupational exposure to industrial chemicals such as aromatic amines (*β*-naphthylamine, 4-aminobiphenyl, 4-chloro-*o*-toluidine and benzidine and 4,4′-methylenebis(2-chloroaniline)) and polycyclic aromatic hydrocarbons (PAHs) have also been associated with bladder cancer [19, 20].

Very few studies have investigated the role of workplace exposure to silica and asbestos in the etiology of bladder cancer. Most published studies reported findings in passing or in analysis that primarily focused on lung cancer, and rarely have investigators assessed exposure-response [1]. The evidence was primarily based on studies using job title or industry as a proxy for exposure. Occupations with an increased risk of bladder cancer include coal miners [21–24], shipyard workers [25], foundry workers [24, 26, 27], chimney sweeps [28], petrochemical workers [29, 30], general labourers [31], textile workers, glass and stone processing, machining and fabricating occupations, excavating, grading, and paving occupations [32] and mechanics and repairers [33] . Others did not observe an overall increased risk of bladder cancer for textile workers in Spain but noted elevated risks among workers with the highest exposures and those working with specific materials or in winding/warping/sizing roles [34, 35]. In a study of marine engineers previously exposed to asbestos, an increased risk of bladder cancer was noted (standardized incidence ratio [SIR] 1.3, 95%CI: 1.0–1.8) when a 40-year lag time was applied [36]. However, a meta-analysis of asbestos-exposed occupational cohorts reported no association [37]. A previous study using NECSS data reported increased bladder cancer risk with self-reported exposure at work to asbestos (odds ratio [OR]: 1.69 95% CI: 1.07–2.65) [30]. However, this earlier analysis used a subset of the NECSS data, including participants from only four of the eight provinces surveyed, and did not use the detailed occupational histories to construct asbestos exposure metrics. In contrast, our expert based assessment reduces exposure misclassification and recall bias and allowed us to consider multiple dimensions of occupational exposure (intensity, duration and frequency).

The purpose of this analysis was to investigate the associations between silica and asbestos exposures at work and bladder cancer using a detailed exposure assessment method that involved professional hygienists who were blinded to case-control status and data from a national population-based case-control study.

## Methods

### Study population

Data for this study were drawn from the case-control component of the NECSS, a collaborative project between Health Canada and cancer registries in eight Canadian provinces. The study design of the NECSS has been previously described [38]. The NECSS recruited incident cancer cases for 19 cancer sites, from provincial cancer registries and cancer-free controls frequency matched on age (5-year groups) and sex to the overall case distribution. Controls were recruited from a random sample of the provincial population obtained from health insurance plans or random-digit dialing depending on the province. The current study was restricted to males, who are more likely to have been occupationally exposed to the agents of interest. A total of 670 bladder cancer cases (66% of those contacted) [31] and 2547 controls (64% of those contacted) [39] completed study questionnaires. Our analysis excluded controls from the province of Ontario as this province did not collect data on bladder cancer cases and was restricted to men ≥40 years of age who had worked for at least 1 year, for a total of 658 histologically confirmed bladder cancer cases and 1360 controls recruited from 7 Canadian provinces.

### Exposure assessment

Questionnaires, mailed in 1994–97, were used to obtain lifetime occupational histories. Participants were asked to provide information for each job held for at least 12 months from the time they were 18 years old to the time of the interview. For each occupation, the information collected included job title, main tasks performed, type of industry, location, period of employment and status (full-, part-time or seasonal). Based on these job descriptions, a team of industrial hygienists carried out a comprehensive exposure assessment to determine the exposure status of each job with respect to asbestos, crystalline silica, diesel emissions, gasoline emissions and aromatic amines using the same method applied by Villeneuve et al. 2011 [40] and described in Sauvé et al. [41]. Only 15 participants overall (< 1%) were assigned exposure to aromatic amines based on job descriptions, primarily to workers in the dyeing industry. Due to the small number of exposed workers, hygienists were only able to assign ever exposure and were not able to assess concentration of exposure to aromatic amines. Based on the very low prevalence of occupational exposure, there is not much concern for potential confounding by aromatic amines in this study population. As in our previously published studies of lung cancer [40, 42], the occupation and industry coding was upgraded to the 7-digit Canadian Classification and Dictionary of Occupation (CCDO) codes (1971–1989) [43]. Controls were coded first, in the context of the aforementioned lung cancer analyses. To ensure consistency when coding the bladder cancer series, job-exposure profiles describing the chemical coding distributions for individual job titles previously assigned to controls were used as general guidelines. The exposure assessment approach involved an expert review by the same team who coded the controls, based on job descriptions, which has previously been described in detail [44, 45]. The assignment of exposures was based on information collected for 12,367 jobs across three dimensions: concentration, frequency, and reliability. Each of these variables was defined using a semi-quantitative scale: none (unexposed), low, medium, or high. Non-exposure was defined as exposure up to background levels found in the general environment. Frequency of exposure was determined based on the proportion of time in a typical workweek that the participant was exposed: low (< 5%), medium (5–30%), and high (> 30%) and was adjusted for part-time and seasonal work. Concentration was assessed on a relative scale with respect to pre-established benchmarks. Low exposure to silica was typically assigned to those employed as construction workers, medium to coal miners and high to sandblasters. For asbestos, low exposure was typically assigned to welders, medium to furnace installers and repairmen and high to asbestos miners. Finally, each exposure was also assigned a reliability value (“possible”, “probable”, or “definite”), estimating the industrial hygienists’ confidence that it was actually present in the job evaluated. We used the reliability score assigned to all exposure values to group those exposures assessed as low reliability with the unexposed. Of the 12,367 jobs, 194 were coded as missing due to incomplete information. A subset of 96 participants with 385 jobs was selected for a reassessment of exposures. Excellent inter-rater agreement was observed for reliability and concentration of exposure on this subset of participants (weighted *κ* = 0.81, 0.78–0.85).

### Exposure metrics

Several metrics were constructed to describe occupational exposure to silica and asbestos including ever exposure, highest attained concentration of exposure, highest attained frequency of exposure, duration of exposure and cumulative exposure. Ever exposure was modeled as a binary variable. Highest attained exposure concentration and frequency of exposure corresponds to the maximum value assigned across all jobs in an individual’s employment history. Duration of exposure was calculated as the number of years employed in jobs where exposure was present and was categorized as tertiles in exposed controls. To estimate cumulative exposure (CE) concentration (C) (low was coded as 1, medium as 5, high as 25), frequency (F) (low: assuming 40 h work week × 5% work-time exposed, medium: 40 h × 15%, high: 40 h × 50%) and duration (D) were combined using the following forumla: CE= ${\sum}_{i=1}^k{C}_i{F}_i{D}_i,$ where i represents the ith job held and k is the total number of jobs held. The transformation of concentration levels to 1, 5 and 25 represented an overall estimate of the relative scale between the semi-quantitative concentration levels assigned by the Montreal industrial hygiene experts across the range of agents [46]. We categorized the continuous measures of CE into tertiles based on the observed frequency distribution in exposed controls. Odds ratios are presented for exposure metrics restricted to probable and definite exposure.

### Other relevant risk factors

The NECSS questionnaire collected information from participants on several additional occupational factors, such as self-reported exposure to 17 chemical substances for more than one year (ever/never). Information on sociodemographic, dietary and behavioral determinants of cancer risk was also collected. This included alcohol consumption, cigarette smoking and cumulative lifetime exposure to secondhand smoke. Dietary information from 2 years prior to the interview was collected using a modified 69-item food-frequency questionnaire (FFQ) that was a combination of the previously validated Block FFQ [47] and Willett instrument used in the Nurses’ Health Study [48]. Furthermore, information on current, past (2 years ago), and seasonal participation in both leisure-time and occupational physical activities was also collected.

### Statistical analysis

Frequencies and percentages were calculated to describe the distribution of variables between cases and controls. Multivariable unconditional logistic regression was used to estimate odds ratios and their corresponding 95% confidence intervals. All models were adjusted for the study design variables of age (10-year categories), proxy respondent status, and province of residence as well as cigarette pack-years, an established bladder cancer risk factor (“minimal” model). We considered additional covariates, such as quartiles of processed meat intake, quartiles of tap water intake, coffee and tea consumption (number of cups/week), quartiles of total and added fat intake, total moderate and total strenuous physical activity (hours/month), education, income and income adequacy (total household gross income/number of individuals supported by this income). Final models were adjusted for variables that changed the effect estimate for ever exposure to silica or asbestos by more than 5% when added to the minimal model. “Full” models were adjusted for highest attained concentration of diesel exposure and ever having worked with mineral/lube oil at work because these factors modified the effect estimate by > 5%. Sensitivity analyses also included lagging silica and asbestos exposure by periods of 20 and 40 years.

## Results

The number of workers exposed and the most common exposure coding (concentration, frequency and reliability) among the 2014 jobs held by participants classified as having probable or certain occupational exposure to crystalline silica and asbestos are presented in Table 1.

**Table 1:** Exposure coding for silica and asbestos among jobs with probable/certain exposure, NECSS 1994–1997

			Most common exposure coding among occupationally exposed (probable or certain)							
			Silica				Asbestos			
CCDO Codes		N (%) jobs	N (%) exposed	Concentration	Frequency	Confidence	N (%) exposed	Concentration	Frequency	Confidence
7111–7199 and 7313–7518	Farming, horticulture, animal husbandry occupations; fishing, forestry, logging and related occupations	376 (18.7)	262 (69.7)	Low (100.0%)	Medium (89.3%)	Probable (100.0%)	0 (0.0)	–	–	–
8780–8799 and 9910–9918	Construction trades and occupations in laboring and elemental work	124 (6.2)	61 (49.2)	Low (86.9%)	Medium (63.9%)	Probable (85.3%)	10 (8.1)	Low (90.0%)	Medium (70.0%)	Probable (90.0%)
8710–8719	Excavating, grading, paving and related occupations in construction	34 (1.7)	27 (79.4)	Low (96.3%)	High (74.1%)	Certain (77.8%)	0 (0.0)	–	–	–
7710–7719	Mining and quarrying including oil and gas field occupations	38 (1.9)	29 (76.3)	Medium (62.1%)	High (89.7%)	Certain (82.8%)	2 (5.3)	Medium (100.0%)	High (100.0%)	Certain (100.0%)
8540–8599 and 8178 and 8230–8290 and 9511–9519	Product fabricating and assembling occupations (wood, rubber, plastic, textiles) and mechanics and repairers	167 (8.3)	14 (9.6)	Low (64.3%)	Medium (92.9%)	Certain (78.6%)	37 (22.2)	Low (97.3%)	Medium (89.2%)	Probable (100.0%)
9111–9199 and 9539	Truck drivers, other transport operating and related occupations	157 (7.8)	9 (5.7)	Low (100.0%)	Medium (66.7%)	Certain (77.8%)	13 (8.3)	Low (100.0%)	Low (92.3%)	Probable (92.3%)
8110–8149 and 8310–8330 and 8510–8529	Mineral ore treating occupations and metal processing and related occupations	29 (1.4)	8 (27.6)	High (75.0%)	High (100.0%)	Certain (100.0%)	0 (0.0)	–	–	–
8150–8165 and 8211	Clay, glass and stone processing, mixing and blending chemicals and related materials	7 (0.4)	0 (0.0)	–	–	–	0 (0.0)	–	–	–
6111–6119, 6120–6199, 8210–8229 and 8293	Protective service occupations, food and beverage preparation and other services occupations	204 (10.1)	0 (0.0)	–	–	–	4 (2.0)	Low (100.0%)	High (50.0%)	Certain (100.0%)
8313–8399	Metal, glass, stone and related materials machining occupations	42 (2.1)	1 (2.4)	Medium (100.0%)	High (100.0%)	Probable (100.0%)	4 (9.5)	Low (50.0%)	Medium (75.0%)	Certain (100.0%)
8731–8739 and 8533–8539	Electrical, lighting and wiring installation and repair	60 (3.0)	3 (5.0)	Low (100.0%)	Low (33.3%)	Probable (66.7%)	23 (38.3)	Low (100.0%)	Medium (95.7%)	Probable (95.7%)
9311–9318	Material handling and related occupations	34 (1.7)	0 (0.0)	–	–	–	1 (2.9)	Medium (100.0%)	High (100.0%)	Definite (100.0%)
9310–9319	Stationary auxiliary and utility equipment operators	28 (1.4)	1 (3.6)				14 (50.0)	Low (100.0%)	Medium (100.0%)	Probable (100.0%)
1111–5199	Office workers, managers, executives, academics and professionals in business, sciences, engineering, teaching, health and arts	576 (28.6)	7 (1.2)	Low (85.7%)	Medium (57.1%)	Probable (71.4%)	0 (0.0)	–	–	–
1000, 2000, 5000, and 9000	Retired, disabled and/or sick, student, or unknown/never worked	138 (6.9)	–	–	–	–	–	–	–	–
	Missing	4								
Total		2014 (100.0)								

Excavating, grading, paving and related occupations in construction had the highest proportion of silica exposed workers (79.4%), followed by mining and quarrying including oil and gas field occupations (76.3%) and farming, horticulture, animal husbandry occupations, fishing, forestry, logging and related occupations (69.7%). Most participants in these occupations were exposed at low concentrations and at medium-high frequencies. Industries with the highest proportion of workers exposed to asbestos included stationary auxiliary and utility equipment operators (50.0%), electrical, lighting and wiring installation and repair (38.3%) and product fabricating and assembling occupations (wood, rubber, plastic, textiles) and mechanics and repairers (22.2%). Most workers were exposed at low concentrations and at a medium frequency.

Select characteristics of the study population are presented in Table 2. Increased odds of bladder cancer were observed with higher cigarette pack-years (p-trend < 0.0001). Bladder cancer cases were more likely to have ever been occupationally exposed to high concentrations of diesel engine emissions (previously reported in [45]), and to have self-reported exposure to mineral/lube oil, welding dust, benzene and benzidine at work. Self-reported exposure to wood dust at work was not related to bladder cancer.

**Table 2:** Select characteristics of bladder cancer cases and controls from the NECSS, 1994–1997

Characteristic	Cases		Controls			
	N	%	N	%	OR ^a^	95% CI
Age at interview						
40- < 50	52	7.9	137	10.1		
50- < 60	126	19.2	239	17.6		
60- < 70	283	43.0	581	42.7		
≥ 70	197	29.9	403	29.6		
Province of residence						
Newfoundland	42	6.4	105	7.7		
Prince Edward Island	15	2.3	63	4.6		
Nova Scotia	60	9.1	307	22.6		
Manitoba	88	13.4	126	9.3		
Saskatchewan	62	9.4	120	8.8		
Alberta	196	29.8	265	19.5		
British Columbia	195	29.6	374	27.5		
Proxy respondent						
No	405	61.6	902	66.3	1.00	
Yes	253	38.5	458	33.7	1.30	1.06–1.59
Cigarette pack-years						
Never smoker	76	11.6	302	22.2	1.00	
> 0- < 10	67	10.2	223	16.4	1.15	0.79–1.68
10- < 20	120	18.2	233	17.1	1.93	1.37–2.72
20- < 30	126	19.2	214	15.7	2.39	1.70–3.38
30- < 40	121	18.4	147	10.8	3.53	2.46–5.07
≥ 40	137	20.8	217	16.0	2.70	1.91–3.81
Unknown	11	1.7	24	1.8	1.72	0.79–3.73
p_-trend_					< 0.001	
Ever exposure to aromatic amines at work						
No	652	99.1	1348	99.1	1.00	
Yes	6	0.9	12	0.9	1.36	0.49–3.79
Highest attained concentration of diesel emissions exposure						
Unexposed	402	61.1	869	63.9	1.00	
Low	162	24.6	377	27.7	0.88	0.70–1.10
Medium	66	10.0	89	6.5	1.46	1.03–2.08
High	28	4.3	25	1.8	2.60	1.47–4.61
p_-trend_					0.007	
Self-reported exposure to wood dust at work						
No	506	76.9	1027	75.5	1.00	
Yes	152	23.1	333	24.5	0.97	0.77–1.21
Self-reported exposure to mineral/lube oil at work						
No	496	75.4	1133	83.3	1.00	
Yes	162	24.6	227	16.7	1.60	1.27–2.03
Self-reported exposure to welding dust at work						
No	490	74.5	1101	81.0	1.00	
Yes	168	25.5	259	19.0	1.44	1.15–1.81
Self-reported exposure to benzene at work						
No	616	93.6	1313	96.5	1.00	
Yes	42	6.4	47	3.5	1.97	1.27–3.07
Self-reported exposure to benzidine at work						
No	639	97.1	1344	98.8	1.00	
Yes	19	2.9	16	1.2	2.62	1.31–5.23
Total	658	100.0	1360	100.0		

^a^Presented odds ratios (OR) are adjusted for age at interview, province of residence, and proxy respondent.

### Silica exposure at work

A total of 254 cases (12.6%) and 431 controls (21.4%) were exposed to silica dust at some point during their working history. In logistic regression models adjusted for age, province of residence, respondent status and cigarette pack-years (minimal model), ever exposure to silica at work was associated with a 29% increase in the odds of bladder cancer (OR:1.29, 95%CI: 1.00–1.61) (Table 3). Restricting ever exposure groups to those ever exposed at least 20 years ago and at least 40 years ago did not change this estimate appreciably. However, further adjustment for highest attained concentration of diesel exposure and self-reported exposure to mineral/lube oil at work (full model) attenuated these estimates. Bladder cancer cases were more likely to have been exposed to low concentrations of silica dust at work than controls (full model OR:1.24, 95%CI: 0.98–1.58). Exposure to medium/high concentrations of silica dust was not related to bladder cancer. High frequency of exposure to silica dust was suggestively associated with bladder cancer as those exposed for 5–30% of work time and more than 30% of work time experienced elevated odds of bladder cancer. Longer duration of exposure (full model OR:1.41, 95%CI: 1.01–1.98) particularly at low concentrations (full model OR: 1.52, 95%CI: 1.07–2.14, p-trend: 0.07) was associated with bladder cancer. Considering concentration, frequency and duration together, slightly increased odds of bladder cancer were observed for those exposed to the lowest and highest tertile of cumulative silica exposure relative to the unexposed.

**Table 3:** Workplace silica exposure and bladder cancer in men from the NECSS, 1994–1997

Silica exposure groups	Cases		Controls		Minimal ^a^	Full ^b^
	N	%	N	%	OR (95% CI)	OR (95% CI)
Ever exposed to silica						
Never	404	20.0	929	46.0	1.00	1.00
Ever	254	12.6	431	21.4	1.27 (1.00–1.61)	1.20 (0.95–1.51)
≥ 20 years ago	57		88		1.29 (0.89–1.88)	1.14 (0.79–1.66)
≥ 40 years ago	146		254		1.21 (0.94–1.55)	1.06 (0.82–1.38)
Highest attained concentration of exposure to silica						
Unexposed	404	20.0	929	46.0	1.00	1.00
Low	218	10.8	369	18.3	1.23 (0.99–1.53)	1.24 (0.98–1.58)
Medium/ High	36	1.8	62	3.1	1.14 (0.73–1.79)	0.96 (0.60–1.54)
p_-trend_					0.05	0.13
Highest attained frequency of exposure to silica						
Unexposed	404	20.0	929	46.0	1.00	1.00
< 5%	18	0.9	51	2.5	0.82 (0.46–1.46)	0.81 (0.45–1.46)
5–30%	160	7.9	274	13.6	1.21 (0.95–1.55)	1.26 (0.97–1.64)
≥ 30%	76	3.8	106	5.3	1.38 (0.99–1.93)	1.22 (0.84–1.77)
p_-trend_					0.03	0.09
Duration of exposure to silica (years)						
Unexposed	404	20.0	929	46.0	1.00	1.00
< 7	78	3.9	134	6.6	1.20 (0.87–1.64)	1.17 (0.84–1.63)
7- < 27	67	3.3	118	5.9	1.12 (0.80–1.57)	1.02 (0.73–1.43)
≥ 27	99	4.9	164	8.1	1.29 (0.96–1.74)	1.41 (1.01–1.98)
Unknown	10	0.5	15	0.7		
p_-trend_					0.07	0.16
Duration of exposure at low concentrations of silica (years)						
Unexposed	421	20.9	968	48.0	1.00	1.00
< 7	75	3.7	124	6.1	1.21 (0.88–1.68)	1.20 (0.86–1.68)
7 - < 27	69	3.4	123	6.1	1.14 (0.82–1.59)	1.09 (0.77–1.55)
≥ 27	83	4.1	132	6.5	1.38 (1.00–1.91)	1.52 (1.07–2.14)
Unknown	10	0.5	13	0.6		
p_-trend_					0.03	0.07
Duration of exposure at medium/high concentrations of silica (years)						
Unexposed	622	30.8	1298	64.3	1.00	1.00
< 7	18	0.9	30	1.5	1.20 (0.65–2.22)	1.07 (0.57–2.00)
≥ 7	18	0.9	30	1.5	1.00 (0.54–1.88)	0.76 (0.39–1.46)
Unknown	0	0.0	2	0.1		
p_-trend_					0.85	0.67
Cumulative exposure to silica						
Unexposed	404	20.0	929	46.0	1.00	1.00
Lowest tertile	85	4.2	132	6.5	1.26 (0.92–1.72)	1.24 (0.90–1.71)
Middle tertile	66	3.3	140	6.9	1.02 (0.73–1.42)	1.03 (0.73–1.46)
Highest tertile	93	4.6	144	7.1	1.35 (0.99–1.83)	1.29 (0.92–1.81)
Unknown	10	0.5	15	0.7		
p_-trend_					0.08	0.18

^a^ Adjusted for province of residence, age at interview, respondent status, cigarette pack-years

^b^ Adjusted for province of residence, age at interview, proxy respondent, cigarette pack-years, highest attained concentration of diesel exposure, ever exposed to mineral/lube oil at work

### Asbestos exposure at work

A total of 120 cases (6.0%) and 151 controls (7.5%) were ever exposed to asbestos in the workplace. In logistic regression models adjusted for age, province of residence, respondent status and cigarette pack-years, ever exposure to asbestos at work, exposure at medium/high concentrations, frequency of exposure of 5–30% of work time, duration of < 10 years at low concentrations and duration of ≥7 years at medium/high concentrations and the lowest tertile of cumulative asbestos exposure were associated with bladder cancer (Table 4). In general, these associations were attenuated in models further adjusted for highest attained concentration of diesel engine emission exposure and ever exposure to mineral/lube oil at work. The results from the fully adjusted model are highlighted. Ever exposure to asbestos at work was associated with a 32% increase in odds of bladder cancer (95%CI: 0.98–1.77). This association was stronger after restricting to those ever exposed at least 20 years ago (OR: 2.04, 95%CI: 1.25–3.34) and attenuated in those ever exposed at least 40 years ago (OR: 1.26, 95%CI: 0.90–1.78). Highest attained concentration of exposure to asbestos was not statistically significantly associated with bladder cancer (p-trend: 0.07). Frequency of exposure for 5–30% of work time was associated with a 45% increase in odds of bladder cancer (OR: 1.45 95%CI: 1.06–1.98). Bladder cancer cases were more likely to have been exposed for durations of < 9 years at any concentration and < 10 years at low concentrations, while duration of exposure at medium/high concentrations was not significantly associated with bladder cancer. Exposure to the lowest tertile of asbestos exposure relative to the unexposed was associated with an increase in odds of bladder cancer (OR: 1.69, 95%CI: 1.07–2.65).

**Table 4:** Workplace asbestos exposure and bladder cancer in men from the NECSS, 1994–1997

Asbestos exposure groups	Cases		Controls		Minimal ^a^	Full ^b^
	N	%	N	%	OR (95% CI)	OR (95% CI)
Ever exposed to asbestos						
Never	538	26.7	1209	59.9	1.00	1.00
Ever	120	6.0	151	7.5	1.58 (1.20–2.08)	1.32 (0.98–1.77)
≥ 20 years ago	44		36		2.51 (1.57–4.03)	2.04 (1.25–3.34)
≥ 40 years ago	84		105		1.64 (1.19–2.25)	1.26 (0.90–1.78)
Highest attained concentration of exposure to asbestos						
Unexposed	538	26.7	1209	59.9	1.00	1.00
Low	106	5.3	134	6.6	1.55 (1.17–2.07)	1.29 (0.95–1.76)
Medium/ High	14	0.7	17	0.8	1.80 (0.85–3.81)	1.56 (0.73–3.32)
p_-trend_					< 0.001	0.07
Highest attained frequency of exposure to asbestos						
Unexposed	538	26.7	1209	59.9	1.00	1.00
< 5%	4	0.2	10	0.5	0.79 (0.24–2.63)	0.63 (0.18–2.15)
5–30%	107	5.3	122	6.1	1.75 (1.31–2.35)	1.45 (1.06–1.98)
≥ 30%	9	0.5	19	0.9	0.92 (0.40–2.10)	0.90 (0.39–2.08)
p_-trend_					< 0.001	0.08
Duration of exposure to asbestos (years)						
Unexposed	538	26.7	1209	59.9	1.00	1.00
< 9	45	2.2	46	2.3	1.90 (1.22–2.95)	1.69 (1.08–2.66)
9 - < 25	39	1.9	51	2.5	1.57 (0.99–2.47)	1.26 (0.78–2.02)
≥ 25	33	1.6	51	2.5	1.27 (0.79–2.03)	1.04 (0.64–1.69)
Unknown	3	0.2	3	0.2		
p_-trend_					< 0.001	0.07
Duration of exposure at low concentrations of asbestos (years)						
Unexposed	547	27.1	1221	60.5	1.00	1.00
< 10	44	2.2	43	2.1	1.98 (1.26–3.11)	1.75 (1.10–2.77)
10 - < 24	33	1.6	44	2.2	1.43 (0.88–2.33)	1.13 (0.68–1.87)
≥ 24	31	1.5	49	2.4	1.30 (0.80–2.10)	1.05 (0.63–1.73)
Unknown	3	0.2	3	0.2		
p_-trend_					< 0.001	0.11
Duration of exposure at medium/high concentrations of asbestos (years)						
Unexposed	644	31.9	1343	66.6	1.00	1.00
< 7	5	0.3	8	0.4	1.61 (0.50–5.19)	1.39 (0.43–4.46)
≥ 7	9	0.5	9	0.5	1.75 (0.66–4.64)	1.54 (0.58–4.14)
p_-trend_					0.14	0.36
Cumulative exposure to asbestos						
Unexposed	538	26.7	1209	59.9	1.00	1.00
Lowest tertile	45	2.2	47	2.3	1.92 (1.24–2.99)	1.69 (1.07–2.65)
Middle tertile	37	1.8	48	2.4	1.47 (0.93–2.34)	1.22 (0.76–1.97)
Highest tertile	35	1.7	53	2.6	1.35 (0.85–2.14)	1.13 (0.70–1.82)
Unknown	3	0.2	3	0.2	0.01	0.23
p_-trend_						

^a^ Adjusted for province of residence, age at interview, proxy respondent and cigarette pack-years

^b^ Adjusted for province of residence, age at interview, proxy respondent, cigarette pack-years, highest attained concentration of diesel exposure, ever exposed to mineral/lube oil at work

### Joint exposure to silica and asbestos at work

Approximately 5% of workers were ever exposed to both silica and asbestos. Ever exposure to both silica and asbestos at work was associated with a 67% increase in the odds of bladder cancer (OR: 1.67, 95%CI: 1.06–2.62) relative to those unexposed to either. Odds ratios for ever exposure to silica but not asbestos and ever exposure to asbestos but not silica were only slightly elevated (Table 5).

**Table 5:** Joint ever exposure to silica and asbestos at work and bladder cancer risk, NECSS 1994–1997

	Cases		Controls		
	N	%	N	%	OR (95% CI) ^a^
Unexposed	335	50.9	832	61.2	1.00
Ever exposed to silica but not asbestos	203	30.9	377	27.7	1.20 (0.93 – 1.54)
Ever exposed to asbestos but not silica	69	10.5	97	7.1	1.33 (0.92 – 1.92)
Ever exposed to both	51	7.8	54	4.0	1.67 (1.06 – 2.62)

^a^ Adjusted for province of residence, age at interview, proxy respondent, cigarette pack-years, highest attained concentration of diesel exposure, ever exposed to mineral/lube oil at work

## Discussion

IARC has classified inhaled crystalline silica (quartz or cristobalite) from occupational sources as a group 1 carcinogen based on evidence of lung carcinogenicity in humans and experimental animals [49, 50]. However, silica carcinogenicity in humans was not detected in all industrial settings. The working group noted that carcinogenicity may depend on the inherent characteristics of the silica particles or on external factors affecting the biological activity or distribution of inhaled particles [50]. Additionally, workers are often exposed to dust mixtures that contain quartz as well as other minerals. Characteristics of the dust particles including size, surface properties, and crystalline form may differ by geological source and industrial processing which can affect the biological activity of the inhaled dust [50].

Several studies have investigated the relationship between bladder cancer and industries and occupations that entail worker exposure to silica or asbestos [21–23, 25, 26, 28, 30, 31, 36, 37, 51]. Many of these were conducted in specialized industrial cohorts and were limited by small numbers of cases and the use of mortality as the outcome, employed crude exposure assessment approaches, relying on job or industry title alone as a proxy for exposure and were limited in their ability to evaluate exposure-response relationships. Additionally, most of the published studies did not include adjustment for confounding by known or suspected risk factors for bladder cancer, thus potential unmeasured confounding is another significant limitation shared by previous epidemiologic studies. As a result, the overall available evidence is inconclusive. Positive associations with bladder cancer have been reported for commercial painters exposed to crystalline silica, asbestos, polycyclic aromatic hydrocarbons, benzene, hexavalent chromium and other agents at work (meta relative risk 1.24 (95%CI: 1.16–1.33 [52];), male chimney sweeps from Sweden, attributed to soot and asbestos with contributions from lifestyle factors (SMR, [28]), female Chinese chrysotile textile workers (SMR, [53]), shipyard workers in Genoa, Italy (SMR, [25]), and roofers and water-proofers potentially exposed to asbestos. However, it was noted that the observed elevated mortality may also have been due to cigarette smoking, exposure to asphalt and coal tar pitch volatiles (PMR, [54]). A population-based case-control study including 15,463 incident cancer cases employed in occupations and industries involving exposure to paints, solvents and textiles reported an excess bladder cancer risk suggesting that exposure to silica carries an increased risk [32]. Other studies did not observe elevated incidence or mortality for occupational exposures to silica and asbestos. No increased incidence of bladder cancer was observed among 40,700 Minnesota (U.S.) taconite mining workers (SIR: 1.0, 95%CI 0.8–1.1) [55], respirable crystalline silica and bladder cancer mortality among workers employed in UK silica sand producing quarries [56], and 3057 male workers employed in an asbestos-cement plant in Northern Israel (SIR, [51]).

We considered latency, concentration, frequency and duration of exposure in our investigation of the role of workplace exposure to silica and asbestos in bladder cancer. In our study, we observed a statistically significant increased risk of bladder cancer for exposure to silica for durations of ≥27 years. Ever exposure to asbestos, particularly for those ever exposed ≥20 years ago, frequency of exposure of 5–30% of work time, duration of exposure of < 9 years at any concentration and < 10 years at low concentrations and the lowest tertile of cumulative asbestos exposure was associated with bladder cancer. Risk of bladder cancer was greater for those ever exposed to both silica and asbestos at work than for those unexposed to either.

Asbestos was widely used as insulation in buildings and as fireproofing from the 1930s to 1980s. Today asbestos is present in insulation and building materials, previously manufactured products and imported asbestos-containing products and continues to be used in industrial construction and commercial sectors (building materials such as shingles, tiles, cement and friction materials such as brake lining and automobile clutch pads) [57]. In addition to the construction industry, asbestos exposures can occur during maintenance, renovation and modification of existing public, residential and commercial buildings. Other occupations where workers are likely exposed to asbestos include brake repair workers and people who repair and maintain ships in the manufacturing industry. Silica exposure is ubiquitous and workers in a number of industries and occupations including grinding, cutting, drilling or chipping are exposed. Most exposure occurs in the construction industry at low and moderate levels among tradespersons and helpers (plumbers, plasterers, bricklayers), heavy equipment operators in a variety of industries, manufacturing and underground mines with limited ventilation [57].

In our study, the results for workplace silica suggest that workers exposed at high frequencies and/or for long durations are at increased risk of bladder cancer. The results for asbestos do not suggest an exposure-response pattern or threshold below which exposure is safe as even low-level exposure seems to be associated with increased risk. It is also possible that the results we observed for asbestos can be explained in part by susceptibility bias [58]. Participants exposed at high concentrations may develop asbestosis or other lung diseases and be removed from occupational exposure. This would affect the estimate of association with bladder cancer which can have latency periods of up to 40 years. It is also possible that due to growing awareness of the harms of asbestos exposure, workers are more protected from exposures where concentrations are known to be high, which may not be the case for workers exposed at low concentrations. These workers may be employed in industries where exposure to asbestos is less obvious such as brake repair mechanics, shipyard workers or those working with imported materials containing asbestos.

It is important to note the limitations of our study to aid in its interpretation. First, the semi-quantitative estimates of exposure assume all subjects within a category are exposed at the same level and that differences in exposure levels are accurately represented by the values assigned to the exposure categories. Variability at work sites is greater than these estimates capture. Potential for exposure measurement error is a further limitation, particularly for exposure estimates of lower confidence. Another limitation is that of reporting error. Inaccuracies in reported job duration, job tasks and other characteristics of the employment may have contributed to misclassification of exposure, possibly more so in the distant past. Furthermore, differential recall of occupational histories between cases and controls may produce recall bias. However, the use of expert assessment helps to reduce this bias [59]. The reliance on proxy respondents for some participants may also have contributed to error in the assessment of exposure and confounders. Villanueva et al. [60] evaluated interviews in a case-control study based on quality (unsatisfactory or questionable, reliable and high quality) and found that higher quality interviews led to stronger associations compared with estimates that did not account for interview quality. This suggests that misclassification of the exposure biased estimates toward the null and consequently excluding unreliable interviews reduced misclassification of exposure in the case-control study. The modest response rates for cases and controls in the NECSS are important to note; however, given that the magnitude and direction of established associations with age and cigarette smoking are as expected, and the lack of association with socioeconomic status, this suggests a minimal impact of selection bias on the reported association estimates. Finally, while our full models are adjusted for highest attained concentration of diesel exhaust at work (expert assessment) and self-reported ever use of mineral lube oil, unmeasured and residual confounding are a potential limitation and it is possible that part of the observed association is due to other correlated occupational carcinogens that were not measured as part of our study.

Despite the limitations listed above, a major strength of this study is the rigorous exposure assessment approach based on detailed lifetime occupational histories. Compared to studies using job title or industry alone, the expert review enhanced our ability to take into consideration idiosyncrasies within each job that can influence exposure dimensions, such as variation in exposure across different industries, time periods and geographic locales. The expert assessment is recognized as the reference method for such a study design [43]. The resulting semi-quantitative indices have been shown to be a credible way of assessing exposure [59], and also serve to mitigate the potential for recall bias that is often introduced in self-reported case-control data. This comprehensive assessment allowed us to investigate different aspects of exposure, such as intensity and duration, and to consider the reliability of these exposure metrics. The comprehensive listing of possible cancer risk factors available in the NECSS permitted adjustment for confounding by other bladder cancer risk factors and some occupational exposures. The availability of a large sample size of incident cases makes for a more informative analysis with more precise estimates of the effects of silica and asbestos exposure. To our knowledge, this is the largest population-based case-control study of silica and asbestos exposure and bladder cancer. The population-based design of the NECSS enabled estimation of risks over a wider range of exposure levels and characterization of the frequency and nature of exposures in the general population. Our results imply a threshold effect for occupational silica exposure but suggest that there is no threshold below which exposure to asbestos is safe.

Additional evidence and replication in independent populations would strengthen the case for increasing prevention efforts specifically targeting silica exposure as a risk factor for bladder cancer. This includes through education and raising awareness of risks among those employed in relevant occupations, which may also encourage the appropriate use of personal protective equipment and workplace measures to reduce exposure. Finally, bladder cancer has a high survival rate if found and treated early, therefore surveillance of workers’ health and screening for those employed in related occupations could lead to early diagnosis and improved treatment outcomes.

## Conclusion

Our findings suggest that both silica and asbestos exposure at work increase the risk of bladder cancer. Results for silica are more consistent with an exposure-response relationship. This study is one of the few that has investigated associations between occupational silica and asbestos exposure and bladder cancer using state-of-the-art exposure characterization. Industrial hygienists assigned exposure to silica and asbestos at an individual level using lifetime occupational histories. The findings from this study inform evidence-based action to enhance prevention efforts, particularly in the industries where workers are regularly exposed.

## Data Availability

The datasets used and/or analyzed during the current study are available from the corresponding author on reasonable request.

## Abbreviations

CCDO: Canadian classification and dictionary of occupation
CE: Cumulative exposure
CI: Confidence interval
IARC: International agency for research on cancer
NECSS: National enhanced cancer surveillance system
OR: Odds ratio
PMR: Proportionate mortality ratio
SIR: Standardized incidence ratio
SMR: Standardized mortality ratio
U.S: United States of America

## References

[1] Guha N, Straif K, Benbrahim-Tallaa L (2011). The IARC monographs on the carcinogenicity of crystalline silica. Med Lav.

[2] Steenland K, Ward E (2014). Silica: a lung carcinogen. CA Cancer J Clin.

[3] CAREX. Silica (Crystalline) Canada: CAREX; 2018 [Available from: https://www.carexcanada.ca/en/silica_(crystalline). Accessed 5 Sept 2018.

[4] OSHA. Workers' Exposure to Respirable Crystalline Silica: Final Rule Overview: U.S. Department of Labor; 2016 [Available from: https://www.osha.gov/Publications/OSHA3683.pdf. Accessed 5 Sept 2018.

[5] Kauppinen T, Toikkanen J, Pedersen D, Young R, Ahrens W, Boffetta P (2000). Occupational exposure to carcinogens in the European Union. Occup Environ Med.

[6] Chen W, Liu Y, Wang H, Hnizdo E, Sun Y, Su L (2012). Long-term exposure to silica dust and risk of total and cause-specific mortality in Chinese workers: a cohort study. PLoS Med.

[7] Poinen-Rughooputh S, Rughooputh MS, Guo Y, Rong Y, Chen W (2016). Occupational exposure to silica dust and risk of lung cancer: an updated meta-analysis of epidemiological studies. BMC Public Health.

[8] Monogrphs I, IARC (2012). Asbestos (Chrysotile, amosite, crocidolite, remolite, actinolite, and anthophyllite).

[9] CAREX. Asbestos Canada: CAREX; 2018 [Available from: http://www.carexcanada.ca/en/asbestos/. Accessed 5 Sept 2018.

[10] Nakane H (2012). Translocation of particles deposited in the respiratory system: a systematic review and statistical analysis. Environ Health Prev Med.

[11] Stuart BO (1984). Deposition and clearance of inhaled particles. Environ Health Perspect.

[12] Ferlay J, Soerjomataram I, Dikshit R, Eser S, Mathers C, Rebelo M (2015). Cancer incidence and mortality worldwide: sources, methods and major patterns in GLOBOCAN 2012. Int J Cancer.

[13] Klaile Y, Schlack K, Boegemann M, Steinestel J, Schrader AJ, Krabbe LM (2016). Variant histology in bladder cancer: how it should change the management in non-muscle invasive and muscle invasive disease?. Transl Androl Urol.

[14] Burger M, Catto JW, Dalbagni G, Grossman HB, Herr H, Karakiewicz P (2013). Epidemiology and risk factors of urothelial bladder cancer. Eur Urol.

[15] Fernandez MI, Lopez JF, Vivaldi B, Coz F (2012). Long-term impact of arsenic in drinking water on bladder cancer health care and mortality rates 20 years after end of exposure. J Urol.

[16] Negri E, La Vecchia C (2001). Epidemiology and prevention of bladder cancer. Eur J Cancer Prev.

[17] Figueroa JD, Koutros S, Colt JS, Kogevinas M, Garcia-Closas M, Real FX, et al. Modification of Occupational Exposures on Bladder Cancer Risk by Common Genetic Polymorphisms. J Natl Cancer Inst. 2015;107(11):djv223. 10.1093/jnci/djv223. 10.1093/jnci/djv223 PMC4675099 26374428

[18] Rushton L, Hutchings S, Brown T (2008). The burden of cancer at work: estimation as the first step to prevention. Occup Environ Med.

[19] Kogevinas M (2003). T Mannetje a, Cordier S, Ranft U, Gonzalez CA, Vineis P, et al. occupation and bladder cancer among men in Western Europe. Cancer Causes Control.

[20] Mitra AP, Cote RJ (2009). Molecular pathogenesis and diagnostics of bladder cancer. Annu Rev Pathol.

[21] Cordier S, Clavel J, Limasset JC, Boccon-Gibod L, Le Moual N, Mandereau L (1993). Occupational risks of bladder cancer in France: a multicentre case-control study. Int J Epidemiol.

[22] Golka K, Bandel T, Schlaefke S, Reich SE, Reckwitz T, Urfer W (1998). Urothelial cancer of the bladder in an area of former coal, iron, and steel industries in Germany: a case-control study. Int J Occup Environ Health.

[23] Schifflers E, Jamart J, Renard V (1987). Tobacco and occupation as risk factors in bladder cancer: a case-control study in southern Belgium. Int J Cancer.

[24] Bosetti C, Boffetta P, La Vecchia C (2007). Occupational exposures to polycyclic aromatic hydrocarbons, and respiratory and urinary tract cancers: a quantitative review to 2005. Ann Oncol.

[25] Puntoni R, Merlo F, Borsa L, Reggiardo G, Garrone E, Ceppi M (2001). A historical cohort mortality study among shipyard workers in Genoa. Italy Am J Ind Med.

[26] Gaertner RR, Theriault GP (2002). Risk of bladder cancer in foundry workers: a meta-analysis. Occup Environ Med.

[27] Sherson D, Svane O, Lynge E (1991). Cancer incidence among foundry workers in Denmark. Arch Environ Health.

[28] Hogstedt C, Jansson C, Hugosson M, Tinnerberg H, Gustavsson P (2013). Cancer incidence in a cohort of Swedish chimney sweeps, 1958-2006. Am J Public Health.

[29] Gun RT, Pratt NL, Griffith EC, Adams GG, Bisby JA, Robinson KL (2004). Update of a prospective study of mortality and cancer incidence in the Australian petroleum industry. Occup Environ Med.

[30] Ugnat AM, Luo W, Semenciw R, Mao Y (2004). Canadian Cancer registries epidemiology research G. occupational exposure to chemical and petrochemical industries and bladder cancer risk in four western Canadian provinces. Chronic Dis Can..

[31] Gaertner RR, Trpeski L, Johnson KC (2004). Canadian Cancer registries epidemiology research G. a case-control study of occupational risk factors for bladder cancer in Canada. Cancer Causes Control.

[32] Band PR, Le ND, MacArthur AC, Fang R, Gallagher RP (2005). Identification of occupational cancer risks in British Columbia: a population-based case-control study of 1129 cases of bladder cancer. J Occup Environ Med.

[33] Samanic CM, Kogevinas M, Silverman DT, Tardon A, Serra C, Malats N (2008). Occupation and bladder cancer in a hospital-based case-control study in Spain. Occup Environ Med.

[34] Serra C, Bonfill X, Sunyer J, Urrutia G, Turuguet D, Bastus R (2000). Bladder cancer in the textile industry. Scand J Work Environ Health.

[35] Serra C, Kogevinas M, Silverman DT, Turuguet D, Tardon A, Garcia-Closas R (2008). Work in the textile industry in Spain and bladder cancer. Occup Environ Med.

[36] Rafnsson V, Sulem P (2003). Cancer incidence among marine engineers, a population-based study (Iceland). Cancer Causes Control.

[37] Goodman M, Morgan RW, Ray R, Malloy CD, Zhao K (1999). Cancer in asbestos-exposed occupational cohorts: a meta-analysis. Cancer Causes Control.

[38] Johnson KC (2000). Status report. National enhanced cancer surveillance system: a federal- provincial collaboration to examine environmental cancer risks. Chronic Dis Can.

[39] Villeneuve PJ, Parent ME, Harris SA, Johnson KC (2012). Canadian Cancer registries epidemiology research G. Occup Expo Asbestos Lung Cancer Men: Evid Popul-Based Case-Control Study Eight Can Provinces BMC Cancer.

[40] Villeneuve PJ, Parent ME, Sahni V, Johnson KC (2011). Canadian Cancer registries epidemiology research G. occupational exposure to diesel and gasoline emissions and lung cancer in Canadian men. Environ Res.

[41] Sauve JF, Lavoue J, Nadon L, Lakhani R, Senhaji Rhazi M, Bourbonnais R (2019). A hybrid expert approach for retrospective assessment of occupational exposures in a population-based case-control study of cancer. Environ Health.

[42] Kachuri L, Villeneuve PJ, Parent ME, Johnson KC (2014). Canadian Cancer registries epidemiology G, Harris SA. Occupational exposure to crystalline silica and the risk of lung cancer in Canadian men. Int J Cancer.

[43] Bouyer J, Hemon D (1993). Comparison of three methods of estimating odds ratios from a job exposure matrix in occupational case-control studies. Am J Epidemiol.

[44] Parent ME, Rousseau MC, Boffetta P, Cohen A, Siemiatycki J (2007). Exposure to diesel and gasoline engine emissions and the risk of lung cancer. Am J Epidemiol.

[45] Latifovic L, Villeneuve PJ, Parent ME, Johnson KC, Kachuri L (2015). The Canadian Cancer registries epidemiology G, et al. bladder cancer and occupational exposure to diesel and gasoline engine emissions among Canadian men. Cancer Med.

[46] Lavoue J, Pintos J, Van Tongeren M, Kincl L, Richardson L, Kauppinen T (2012). Comparison of exposure estimates in the Finnish job-exposure matrix FINJEM with a JEM derived from expert assessments performed in Montreal. Occup Environ Med.

[47] Block G, Hartman AM, Naughton D (1990). A reduced dietary questionnaire: development and validation. Epidemiology.

[48] Willett WC (1998). Invited commentary: comparison of food frequency questionnaires. Am J Epidemiol.

[49] Steenland K, Mannetje A, Boffetta P, Stayner L, Attfield M, Chen J (2001). Pooled exposure-response analyses and risk assessment for lung cancer in 10 cohorts of silica-exposed workers: an IARC multicentre study. Cancer Causes Control.

[50] IARC (1997). Silica dust, crystalline, in the form of quartz or cristobalite. In: Monogrphs I, editor.

[51] Tulchinsky TH, Ginsberg GM, Iscovich J, Shihab S, Fischbein A, Richter ED (1999). Cancer in ex-asbestos cement workers in Israel, 1953-1992. Am J Ind Med.

[52] Myong JP, Cho Y, Choi M, Kim HR (2018). Overview of occupational cancer in painters in Korea. Ann Occup Environ Med.

[53] Wang X, Lin S, Yu I, Qiu H, Lan Y, Yano E (2013). Cause-specific mortality in a Chinese chrysotile textile worker cohort. Cancer Sci.

[54] Stern FB, Ruder AM, Chen G (2000). Proportionate mortality among unionized roofers and waterproofers. Am J Ind Med.

[55] Allen EM, Alexander BH, MacLehose RF, Nelson HH, Ramachandran G, Mandel JH (2015). Cancer incidence among Minnesota taconite mining industry workers. Ann Epidemiol.

[56] Brown TP, Rushton L (2005). Mortality in the UK industrial silica sand industry: 2. A retrospective cohort study. Occup Environ Med.

[57] OCRC C. In: OCR C, editor. Burden of Occupational Cancer in Ontario. Major workplace carcinogens and prevention of exposure. Toronto: Ontario Health and Occupational Cancer Research Centre; 2017. http://www.occupationalcancer.ca/wp-content/uploads/2017/09/Burden-of-Occupational-Cancer-in-Ontario.pdf. Accessed 5 Sept 2018.

[58] Horwitz RI, McFarlane MJ, Brennan TA, Feinstein AR (1985). The role of susceptibility bias in epidemiologic research. Arch Intern Med.

[59] Teschke K (2003). Exposure surrogates: job-exposure matrices, self-reports, and expert evaluations. Chap..

[60] Villanueva CM, Silverman DT, Malats N, Tardon A, Garcia-Closas R, Serra C (2009). Determinants of quality of interview and impact on risk estimates in a case-control study of bladder cancer. Am J Epidemiol.

